# Coping Strategies Among Healthcare Workers During the COVID-19 Pandemic: Emotional Responses, Challenges, and Adaptive Practices

**DOI:** 10.3390/medicina61020311

**Published:** 2025-02-11

**Authors:** Aida Puia, Sorina Rodica Pop, Bianca Olivia Cojan Manzat, Sebastian Pintea, Ion Cosmin Puia, Mihaela Fadgyas-Stanculete

**Affiliations:** 1 Department of Community Medicine, “Iuliu Hațieganu” University of Medicine and Pharmacy, 400347 Cluj-Napoca, Romania; aida.puia@umfcluj.ro (A.P.); minzat.bianca@elearn.umfcluj.ro (B.O.C.M.); 2 Department of Psychology, Babeș-Bolyai University, 400084 Cluj-Napoca, Romania; sebastianpintea@psychology.ro; 3 Regional Institute of Gastroenterology and Hepatology “Octavian Fodor”, 400394 Cluj-Napoca, Romania; cosmin.puia@umfcluj.ro; 4 Department of Surgery, “Iuliu Hatieganu” University of Medicine and Pharmacy, 400012 Cluj-Napoca, Romania; 5 Department of Neurosciences, Discipline of Psychiatry and Pediatric Psychiatry, “Iuliu Hatieganu” University of Medicine and Pharmacy, 400012 Cluj-Napoca, Romania; mihaela.fadgyas@umfcluj.ro

**Keywords:** healthcare workers, COVID-19, psychological distress, coping mechanisms, mental health

## Abstract

*Background and Objectives*: The COVID-19 pandemic has posed unprecedented challenges to healthcare workers, leading to significant psychological distress, altered health-related behaviors, and reliance on various coping mechanisms. Understanding these impacts is critical for developing targeted interventions to support healthcare professionals. This study aimed to evaluate the psychological stressors, emotional responses, changes in healthy behaviors, and coping mechanisms employed by healthcare workers during the COVID-19 pandemic. The study further examined differences across demographic and professional groups and explored correlations between stressors, coping strategies, and emotional outcomes. *Materials and Methods*: A cross-sectional survey was conducted among 338 healthcare workers, including physicians and nurses, in urban and rural healthcare settings during the pandemic. Data were collected using validated instruments to measure emotional responses (anxiety and anger), lifestyle behaviors (dietary habits, sleep patterns, physical activity, and smoking), and coping strategies. Statistical analyses included descriptive, inferential, and correlation techniques to assess relationships between variables. *Results*: Fear of infecting family members (M = 3.36, SD = 0.86) and concerns about inadequate protective equipment (M = 2.80, SD = 0.95) were the most significant stressors, strongly associated with heightened anxiety and anger. Changes in healthy behaviors were observed: 69.2% maintained a healthy meal schedule, 56.5% reported disrupted sleep patterns, and only 39.6% engaged in regular physical activity. Among smokers (27.5%), 31.1% increased smoking as a maladaptive coping strategy, while 21.1% reduced smoking. Nurses predominantly relied on emotion-focused strategies, such as religious coping and venting, whereas physicians favored problem-focused strategies like planning and active coping. Social support emerged as a protective factor, mitigating stress and facilitating adaptive coping. *Conclusions*: The study revealed significant psychological and behavioral impacts on healthcare workers during the COVID-19 pandemic. Key stressors included the fear of infecting family members, concerns about inadequate protective measures, and the prolonged uncertainty of the pandemic, which contributed to heightened levels of anxiety and anger. Changes in healthy behaviors, such as disrupted sleep patterns, decreased physical activity, and increased reliance on maladaptive coping mechanisms, further underscored the multifaceted challenges faced by healthcare professionals. Although the acute phase of the pandemic has passed, the long-term consequences on the mental health and well-being of healthcare workers remain critical concerns. Further research is essential to develop effective strategies for monitoring, preventing, and addressing psychological distress among healthcare professionals, ensuring their preparedness for future public health crises.

## 1. Introduction

Healthcare workers have consistently been at the forefront of the pandemic response, bearing the brunt of physical, psychological, and emotional challenges. Historical evidence has highlighted their increased vulnerability to psychological distress during pandemics [1]. Systematic reviews and meta-analyses have demonstrated that healthcare personnel exposed to virus-related work environments are 1.7 times more likely to develop psychological distress and post-traumatic stress disorder (PTSD) than their non-exposed counterparts [2,3]. This vulnerability is attributed to several common risk factors, including the fear of personal safety, uncertainty due to rapidly evolving information, and the threat of societal discrimination. These stressors were evident during earlier pandemics, such as SARS, in which healthcare workers reported elevated levels of anxiety, depression, and burnout during and after the outbreak [4].

Burnout, a recurring theme in studies of healthcare personnel during pandemics, reflects intense pressure on their work environments [5]. During the SARS outbreak, nursing staff, physicians, and healthcare assistants experienced significant distress levels, as documented by Khasne et al. [6]. The COVID-19 pandemic has amplified these challenges owing to its prolonged nature and unprecedented global scale. Several authors noted that this pandemic introduced new levels of workload, resource constraints, and emotional demands, leading to burnout rates surpassing those observed during SARS and MERS [7,8]. Furthermore, the mental health burden is not limited to frontline workers. Non-frontline healthcare workers reported comparable levels of anxiety, depression, and perceived stress [9]. These findings underscore the pervasive psychological toll of pandemics on healthcare personnel across various roles and settings.

One of the most notable lifestyle changes among healthcare workers during the pandemic has been a decline in physical activity. Jin et al. reported that many healthcare workers experienced a decrease in physical activity levels, which was associated with increased anxiety and depression [10]. This finding aligns with research indicating that a sedentary lifestyle can exacerbate mental health issues, particularly during stressful periods such as the COVID-19 pandemic [11]. The constraints imposed by long working hours and the need for personal protective equipment (PPE) have further limited opportunities for physical exercise, contributing to a more sedentary lifestyle among healthcare professionals [12].

In addition to reduced physical activity, dietary habits have also been adversely affected. Renzo et al. found that a significant proportion of respondents reported changes in their eating habits during the lockdown, with many adopting unhealthier dietary patterns [13]. This shift toward poor nutrition can have detrimental effects on overall health and immunity, which is particularly concerning given the ongoing health crisis. The stress and emotional toll of working in high-pressure environments have led some healthcare workers to engage in unhealthy eating behaviors as a coping mechanism, further compounding the challenges they face [14].

Beyond the medical field, elevated levels of anxiety and fluctuations in well-being have also been observed in the education sector, as evidenced by recent studies on academic engagement and stress among university students [15,16].

The interplay among coping mechanisms, lifestyle habits, and perceived support systems plays a crucial role in determining healthcare workers’ resilience to such stressors [17,18]. Adaptive coping strategies, healthy lifestyle behaviors, and robust social and organizational support systems are critical for maintaining psychological well-being. However, the COVID-19 pandemic has disrupted these dimensions, potentially influencing healthcare workers’ capacity to effectively manage stress [19]. Although the literature provides valuable insights into these individual factors, comprehensive research exploring their interconnections remains limited.

This study aimed to address this gap by adopting a multidimensional approach to examine the impact of the COVID-19 pandemic on healthcare workers’ coping mechanisms, lifestyle habits, and perceived support systems. Unlike previous studies that focused on isolated aspects, this study investigates the collective influence of these factors to provide a holistic understanding of how healthcare workers adapted during the pandemic.

Additionally, this study included a diverse sample of healthcare workers, encompassing both frontline and non-frontline staff across various roles and settings. This diversity allows for a nuanced analysis of how stressor exposure, occupational context, and resource availability influence coping strategies and perceived support during the pandemic. The research also explored the role of specific organizational and social support mechanisms in mitigating stress and promoting resilience.

By addressing these unique aspects, this study seeks to contribute novel insights to the field, advancing both theoretical understanding and practical strategies for supporting healthcare workers. These findings aim to inform targeted interventions and policies to enhance resilience and well-being among healthcare personnel both in the context of ongoing public health crises and for future preparedness efforts.

## 2. Materials and Methods

### 2.1. The Study Design and Participants

This cross-sectional study investigated psychosocial factors influencing emotional distress and coping mechanisms among healthcare personnel in Romania during the COVID-19 pandemic. Participants included physicians and nurses working in various healthcare settings. Individuals were recruited online through professional networks and institutional emails between May 2020 and June 2020.

Out of 500 invited participants, 338 completed the survey, yielding a response rate of 67.6%. We used multiple imputations for continuous variables, employing predictive mean matching (PMM) to replace missing values based on observed data distributions. Categorical variables with more than 5% missing values were excluded from the analysis using listwise deletion.

### 2.2. Study Procedure

This study was conducted online to ensure accessibility and adhere to pandemic-related restrictions. After providing informed consent, participants completed a structured survey via a secure online platform. The survey comprised validated psychological scales and a demographic questionnaire.

#### Measures

1. Demographic Questionnaire: This section collected information on age, sex, educational level, professional role, years of experience, work setting (e.g., hospital, clinic), and exposure to COVID-19 patients.
2. COVID-19 Stressor Questionnaire: This instrument, developed for this study, includes items that address the key themes highlighted in studies of previous SARS outbreaks and the initial stages of the COVID-19 pandemic in China:
  - Changes in work schedules and responsibilities;
  - Exposure to infected patients and personal protective equipment (PPE) use;
  - Isolation, quarantine, and hospitalization experiences.

Validation of the COVID-19 Stressor Questionnaire: The COVID-19 Stressor Questionnaire was adapted from validated pandemic-related stressor scales used in previous SARS and MERS research. The instrument underwent pilot testing with 30 healthcare workers to ensure reliability. Cronbach’s alpha for internal consistency was 0.87. Construct validity was assessed via exploratory factor analysis, confirming distinct factors related to occupational, health, and personal stressors.

Power Analysis: A power analysis was conducted using G*Power (v3.1) to determine the required sample size. Assuming a moderate effect size (Cohen’s d = 0.5) and 80% power with a significance level of 0.05, the minimum required sample size was 300. Our final sample (N = 338) exceeded this threshold, supporting adequate statistical power.

3. Brief-COPE Scale: This 28-item validated scale [20] measured participants’ coping strategies, identifying adaptive (e.g., problem-solving, Positive Reframing) and maladaptive strategies (e.g., denial, substance use).
4. Spielberger State Anxiety Inventory (STAI-s): This scale [21] assessed state anxiety, reflecting immediate emotional responses to stressors. Participants rated responses on a 4-point Likert scale, with higher scores indicating greater anxiety.
5. Spielberger State Anger Inventory (STAXI-s): This instrument [22] evaluated anger levels in response to pandemic-related stressors, including the intensity and frequency of anger.

### 2.3. Ethical Considerations

This study adhered to the ethical guidelines of the Declaration of Helsinki. Ethical approval was obtained from [Ethical Committee of the College of Physicians Cluj nr. 1056/7 May 2020] prior to data collection. Participation was voluntary, and all data were anonymized to ensure confidentiality. Participants could withdraw at any time without penalty.

### 2.4. Data Analysis

Statistical Analysis

The dataset was analyzed using robust statistical techniques to ensure comprehensive insights into the psychological, emotional, and behavioral responses of healthcare workers during the COVID-19 pandemic.

1. Descriptive Statistics: Frequencies and percentages summarized demographic variables, COVID-19 exposure, and lifestyle changes. Means (M) and standard deviations (SD) were used to describe the central tendencies and variability of stressors, coping strategies, and emotional responses.
2. Inferential Statistics:
  - Independent *t*-tests: Assessed differences in anxiety and anger levels between professional groups (physicians vs. nurses);
  - One-way ANOVA: Explored variations in emotional responses and coping strategies across age groups;
  - Pearson’s Correlation Coefficients: Evaluated relationships between stressors, coping strategies, and emotional responses;
  - Biserial Correlations: Analyzed the impact of binary COVID-19 exposure events on emotional responses.
3. Significance Thresholds: Statistical significance was determined at *p* < 0.05, with results at *p* < 0.01 highlighted for stronger associations.

All statistical significance values are consistently reported as *p*-values (e.g., *p* < 0.05).

Definition of Correlation Strength Categories: small: 0.10 ≤ r < 0.30, moderate: 0.30 ≤ r < 0.50, strong: r ≥ 0.50.

## 3. Results

Demographic Overview

The sample included 338 participants, predominantly female (79.3%) and residing in urban areas (83.7%). The age distribution ranged from 20 to 70 years, with the majority in the 40–49 age group (30.8%). Professionally, 39.3% were nurses, and 60.7% were physicians.

Table 1 provides an overview of the demographic characteristics of the participants, including details on age, gender, profession, and residential area.

Table 2 presents the distribution of variables related to participants’ lifestyles, highlighting key factors such as dietary habits, physical activity, and sleep patterns during the pandemic.

Lifestyle behaviors during the pandemic revealed several notable changes. Many participants (69.2%) reported maintaining a healthy meal schedule, while 56.5% experienced changes in their sleep patterns. Of those, most (56.5%) averaged 6–8 h of sleep per night. Regular physical activity also declined, with only 39.6% of participants engaging in exercise during this period.

COVID-19 Exposure and Emotional Responses

Table 3 presents the distribution of variables related to participants’ exposure during the pandemic, highlighting factors such as direct contact with infected individuals, work environment, and personal experiences.

Direct or Indirect Exposure:

In terms of exposure, 49.1% of participants reported direct contact with confirmed or suspected COVID-19 cases, while 29.6% experienced indirect exposure. However, neither direct nor indirect exposure was significantly associated with higher levels of anger or anxiety (*p* > 0.05). Additionally, recommendations for testing and isolation did not show a substantial link to changes in emotional states.

Key psychological stressors were identified:

Table 4 presents the hierarchy of factors perceived as triggers of stress among healthcare workers during the pandemic based on the mean scores of participants’ responses. The most significant stress trigger was the thought of potentially infecting family members, followed by concerns about minor errors leading to infection. Uncertainty about the pandemic’s duration and the lack of specific treatment for the disease also ranked highly. In contrast, the perception of ineffective hospital measures to prevent COVID-19 spread and mild respiratory symptoms causing fear of infection were ranked lower.

Emotional Responses

Anger:

Anger levels were similar across professional groups, with physicians reporting a mean score of 17.90 and nurses reporting a mean score of 18.32, showing no significant difference (*p* > 0.05). Age was not a significant factor in influencing anger levels (*p* > 0.05).

Anxiety:

Anxiety levels were also consistent across professions, with physicians having a mean score of 41.60 and nurses 41.32 (*p* > 0.05). However, prolonged exposure to pandemic-related news (≥4 h/day) was associated with higher anxiety levels, with those exposed reporting a mean score of 45.36 (*p* = 0.056).

Coping Strategies

Coping Strategies by Profession

The study examined various coping strategies employed by healthcare workers, comparing physicians and nurses. The results revealed both similarities and differences between the two professional groups.

Significant Differences:

1. Acceptance: Physicians reported significantly higher use of acceptance as a coping strategy (M = 2.40, SD = 0.61) compared to nurses (M = 2.24, SD = 0.79), with a statistically significant difference (t = 2.06, *p* = 0.041);
2. Religion: Nurses were more likely to use religion as a coping mechanism (M = 1.86, SD = 1.01) compared to physicians (M = 1.38, SD = 1.05), with a strong statistical difference (t = −4.21, *p* = 0.000);
3. Using Emotional Support: Nurses reported using emotional support more frequently (M = 1.75, SD = 1.00) than physicians (M = 1.48, SD = 0.90), with a significant difference (t = −2.61, *p* = 0.010);
4. Venting: Nurses also reported higher use of venting (M = 1.06, SD = 0.85) compared to physicians (M = 0.80, SD = 0.72), with a statistically significant difference (t = −2.19, *p* = 0.029);
5. Substance Use: Physicians were more likely to engage in substance use (M **=** 0.30, SD = 0.63) compared to nurses (M = 0.12, SD = 0.45), with a significant difference (t = 2.98, *p* = 0.003);
6. Denial: Nurses reported significantly higher use of denial (M = 0.59, SD = 0.87) compared to physicians (M = 0.25, SD = 0.55), with a strong statistical difference (t = −4.37, *p* = 0.000).

Non-Significant Differences:

Active Coping, Planning, Positive Reframing, Humor, Using Instrumental Support, Self-Distraction, Behavioral Disengagement, Self-Blame, Problem-Focused Coping, Emotional Coping, and Avoidance Coping showed no significant differences between physicians and nurses (*p* > 0.05).

Age differences in coping:

Older participants (50–70 years) utilized active coping significantly more than their younger counterparts (F = 8.853, *p* < 0.001).

The following table (Table 5) presents the coping strategies employed by physicians and nurses during the pandemic, highlighting both significant and non-significant differences between the two groups. The table includes the mean (M), standard deviation (SD), t-values, and *p*-values for each strategy.

Emotional Responses

The emotional responses, specifically anger and anxiety, were measured to understand the psychological impact of the pandemic. No significant differences in anger or anxiety levels were found between physicians and nurses, indicating that both groups experienced similar emotional reactions to pandemic-related stressors.

Anger: The mean anger scores were comparable across professional groups, with physicians reporting an average score of 17.90 and nurses having an average score of 18.32, showing no significant difference (*p* > 0.05). Age also did not significantly influence anger levels (*p* > 0.05).

Anxiety: Anxiety levels were consistent across both professions, with physicians reporting a mean score of 41.60 and nurses a mean score of 41.32 (*p* > 0.05). However, prolonged exposure to pandemic-related news (≥4 h/day) was associated with higher anxiety levels, with those exposed reporting a mean score of 45.36 (*p* = 0.056).

Correlations Between Coping Strategies and Emotional Responses

Correlations were examined to explore the relationships between coping strategies and emotional responses. Several coping strategies showed significant correlations with emotional states, such as the following:

Venting: A strong positive correlation with anger (r = 0.36) and anxiety (r = 0.42) was found, indicating that individuals who engaged in venting were more likely to experience heightened emotional distress.

Substance Use: A moderate positive correlation with anger (r = 0.26) and anxiety (r = 0.22) was observed, suggesting that higher levels of substance use were linked to increased emotional distress.

Active Coping: A moderate positive correlation with anxiety (r = 0.29) and anger (r = 0.14) suggests that active coping strategies were associated with increased emotional regulation, though not always effective in reducing negative emotions.

Positive Reframing: A strong negative correlation with anxiety (r = −0.24) and anger (r = −0.21), indicating that those who employed Positive Reframing as a coping strategy tended to experience lower levels of anxiety and anger.

Self-Distraction: A moderate positive correlation with anger (r = 0.15) and anxiety (r = 0.21), suggesting that using distraction could help manage both anger and anxiety in certain cases.

Self-Blame: A strong positive correlation with anxiety (r = 0.41) and anger (r = 0.20), indicating that individuals who blamed themselves were more likely to report higher levels of emotional distress.

Overall, the results underscore the variability in coping strategies among healthcare workers and their association with emotional outcomes. Some strategies, such as Positive Reframing, helped to reduce emotional distress, while others, like Venting and Substance Use, were associated with greater emotional difficulties.

Maladaptive strategies such as denial and self-blame were associated with heightened anger and anxiety (*p* < 0.01).

Stressors, such as fear of infecting family members and inadequate protective measures, were positively correlated with anxiety (r = 0.36, *p* < 0.01) and anger (r = 0.19, *p* < 0.01).

Personal Events Related to COVID−19 and Their Correlation with Anger and Anxiety

Table 6 presents the biserial correlations between various personal events related to COVID-19 (coded as Yes = 1, No = 0) and the levels of anger and anxiety experienced by healthcare workers. This table explores the relationships between specific events—such as close contact with confirmed or suspected COVID-19 cases, isolation recommendations, and COVID-19 testing—and emotional responses such as anger and anxiety.

The analysis revealed that close contact with confirmed or suspected COVID-19 cases did not exhibit a significant correlation with anger or anxiety. Similarly, being classified as indirect contact with a confirmed case did not demonstrate a significant association with these emotional responses. Contact with family members during the period of potential exposure showed a moderate positive correlation with anger, although it did not reach statistical significance for anxiety. Additionally, isolation recommendations following exposure to a suspected or confirmed case were not significantly correlated with anger or anxiety. Finally, COVID-19 testing did not show a significant correlation with anger; however, a small negative correlation with anxiety was observed, suggesting a potential, though not statistically significant, marginal alleviation of anxiety following testing.

## 4. Discussion

This study investigated the multifaceted impact of the COVID-19 pandemic on healthcare workers, examining psychological well-being, coping mechanisms, and health-related behaviors. Our findings contribute to a growing body of literature documenting the profound effects of the pandemic on this essential workforce while also highlighting opportunities for targeted support and intervention.

### 4.1. Psychological Distress Among Healthcare Workers

**Figure 1:**
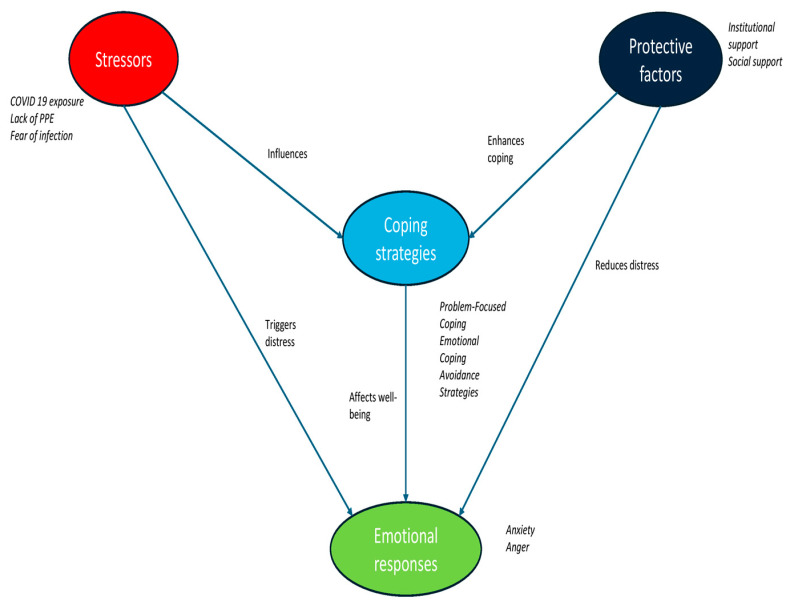
Conceptual framework of stressors, coping strategies, and emotional responses.

A conceptual framework (Figure 1) visually represents the relationships between stressors, coping strategies, and emotional responses. The model highlights how different stressors, such as COVID-19 exposure and workload pressure, influence coping mechanisms, which in turn affect emotional responses like anxiety and anger. These findings align with existing research, underscoring the importance of institutional interventions to support adaptive coping.

Consistent with previous research [7,9], fear of infecting family members emerged as a salient stressor for healthcare workers, strongly associated with heightened anxiety. This underscores the unique burden placed upon healthcare professionals who, while caring for the sick, also grapple with the potential of transmitting the virus to their loved ones. Concerns about inadequate personal protective equipment (PPE) further exacerbated anxiety and anger, echoing findings by Busch et al. on the importance of perceived safety in mitigating distress [23]. The uncertainty surrounding the pandemic’s duration added another layer of psychological complexity, contributing to an overall sense of prolonged stress and apprehension [4].

### 4.2. Changes in Healthy Behaviors

The pandemic significantly disrupted the lifestyle habits and health-related behaviors of healthcare workers. While the majority (69.2%) maintained a healthy meal schedule, demonstrating resilience amidst challenging circumstances, 30.8% reported deviations, potentially attributable to time constraints, fatigue, and limited access to healthy food options, as observed by Tabur et al. [24]. Disrupted sleep patterns were prevalent, with 56.5% of participants reporting compromised sleep quality likely due to anxiety, irregular work hours, and emotional distress, consistent with Shi et al. [25]. Furthermore, only 39.6% of participants engaged in regular physical activity, reflecting a decline potentially driven by increased workloads and restricted access to gyms, mirroring trends reported by other studies [26,27]. Among smokers (27.5%), we observed a dichotomy: 31.1% reported increased tobacco use, suggesting reliance on maladaptive coping mechanisms, while 21.1% reduced their smoking, possibly indicating heightened health awareness during the pandemic. These patterns align with other findings that noted an increase in substance use as a response to stress [28].

### 4.3. Positive Correlation Between Active Coping and Anxiety

An unanticipated finding of this study is the positive correlation between active coping and anxiety. While active coping is generally considered an adaptive strategy, in high-stress environments, such as the COVID-19 pandemic, it may paradoxically contribute to increased anxiety. This phenomenon can be attributed to the substantial emotional and cognitive demands required to actively manage stressors in uncertain situations. Healthcare workers who employ active coping strategies may experience greater distress if their efforts do not yield anticipated control over the situation. Comparable findings have been reported in other high-stakes professions, where excessive reliance on active coping can exacerbate rather than alleviate stress [29,30]. Further research is warranted to investigate whether resilience training or psychological support mitigates this effect.

### 4.4. Urban vs. Rural Differences in Coping Mechanisms

This study observed differences in coping mechanisms between urban and rural healthcare workers. Urban healthcare workers demonstrated a tendency to utilize problem-focused coping strategies, potentially attributable to greater institutional resources, access to mental health services, and more robust professional support networks. In contrast, rural healthcare workers exhibited a higher reliance on emotion-focused coping strategies, such as religious practices and emotional expression, which may be attributed to limited access to mental health resources and increased isolation from professional support systems. These findings suggest that interventions designed to support healthcare workers should be tailored to their geographic context, with enhanced provision of psychological resources in rural settings.

Religious coping strategies were particularly prominent among nurses in our study, who sought comfort and strength through faith. This aligns with other research, which suggests that religiosity can serve as a buffer against stress and depression [29,30,31]. While religious coping can provide a sense of purpose and emotional relief, it is crucial to recognize that over-reliance on emotion-focused coping strategies, such as venting, was associated with heightened distress in our study. This underscores the need for balanced coping mechanisms that incorporate both emotional support and problem-solving approaches.

### 4.5. Impact of Stressors on Behavior and Performance

The demanding work environment during the pandemic, characterized by long working hours and intense pressure to perform under extreme conditions, contributed to increased burnout [29]. The fear of transmitting the virus to loved ones further compounded emotional distress, potentially creating a detrimental cycle impacting both mental health and job performance, as reported by other studies [32,33,34]. These findings highlight the importance of addressing occupational demands and providing comprehensive psychological support to maintain the well-being of healthcare workers and ensure the quality of patient care.

### 4.6. Organizational Support and Interventions

Our findings underscore the critical role of institutional support in mitigating pandemic-related stressors. Effective communication, access to mental health resources, and a supportive work environment were identified as crucial factors in alleviating psychological burdens. These findings align with previous studies, which emphasized the importance of organizational interventions in promoting mental well-being [35,36,37,38].

This study builds upon existing research by incorporating a diverse participant sample encompassing both frontline and non-frontline healthcare workers across urban and rural settings. This diversity allowed for a nuanced analysis of how occupational context and demographic factors influence psychological responses, coping mechanisms, and lifestyle behaviors. Future research should employ longitudinal designs to explore the long-term effects of public health crises on healthcare workers’ psychological and physical health. Mixed-method approaches, including qualitative interviews, could provide deeper insights into healthcare workers’ lived experiences. Furthermore, evaluating the efficacy of specific interventions, such as structured social support programs and workplace adjustments, is essential for informing evidence-based practices. By understanding the complex interplay of individual, organizational, and societal factors, we can develop comprehensive strategies to support the well-being of healthcare workers during and after public health emergencies.

### 4.7. Limitations and Implications for Result Interpretation

This study, while providing valuable insights into the psychological and behavioral responses of healthcare professionals during the COVID-19 pandemic, is subject to several limitations that warrant acknowledgment, along with their potential implications for interpreting the results.

The study sample comprised predominantly female participants (79.3%), which may limit the generalizability of the findings to male healthcare workers or sex-diverse groups. This gender imbalance may have influenced the interpretation of the results, as gender can affect psychological responses and coping strategies. Furthermore, the sample was drawn from a specific geographical and institutional context, potentially limiting the applicability of the results to other regions or healthcare systems with different resources, protocols, and cultural dynamics. The specific context of the study may have influenced the observed patterns of stressors, coping mechanisms, and emotional responses, thereby challenging the generalizability of the findings to other settings.

The cross-sectional nature of the study captures a snapshot of psychological and coping responses at a specific point during the pandemic. This design precludes the examination of changes over time and the long-term psychological effects of prolonged exposure to pandemic-related stressors. These results may not reflect the dynamic and evolving nature of psychological responses and coping strategies over the course of the pandemic. Additionally, this study’s cross-sectional design limits its ability to draw causal conclusions. The observed associations between variables may not necessarily indicate causal relationships, as other unmeasured factors could influence the observed patterns.

The reliance on self-reported questionnaires introduces the possibility of response bias, such as social desirability bias, wherein participants may underreport negative emotions or maladaptive coping mechanisms. This potential bias may result in an underestimation of the true prevalence of psychological distress or maladaptive coping strategies among healthcare workers.

While the study focused on key stressors, emotional responses, and coping strategies, other potentially influential variables, such as pre-existing mental health conditions, personal loss during the pandemic, and support received from family and friends, were not accounted for and could affect the findings. These unmeasured variables may confound the observed relationships among the study variables, making it challenging to isolate the specific effects of the factors under investigation. Additionally, this study did not explore the role of institutional factors, such as workplace policies, leadership support, or availability of resources, in shaping psychological responses and coping behaviors. These findings may not fully reflect the influence of institutional factors on the psychological well-being and coping strategies of healthcare workers.

The study utilized standardized scales and predefined categories for coping strategies and emotional responses, which, while useful for comparability, may not fully capture the nuanced and evolving nature of individual experiences during a pandemic. The use of standardized measures may limit the ability to capture the full range and complexity of individual responses to the pandemic.

The findings of this investigation were constrained by the specific sample and context examined, necessitating cautious extrapolation to alternative settings or populations. To address these limitations, future research endeavors could adopt longitudinal methodologies to monitor temporal shifts in psychological responses and coping mechanisms, encompass diverse participant cohorts across varied geographical and cultural landscapes, and explore additional factors that influence stress and resilience. Furthermore, the incorporation of mixed-method approaches such as in-depth qualitative interviews could yield a more nuanced comprehension of healthcare professionals’ lived experiences and the contextual elements shaping their reactions to public health emergencies.

This study has several strengths that enhance its relevance and impact. Conducted during a critical phase of the COVID-19 pandemic, it focuses on healthcare professionals—a high-risk population—providing valuable insights into their unique stressors and coping mechanisms. The comprehensive design integrates demographic variables, emotional responses, and coping strategies, offering a nuanced understanding of the psychological impact.

The diverse participant sample, including various age groups, professions, and residential settings, enhances the applicability of the findings. Methodological rigor, through validated instruments and robust statistical analyses, ensures reliable results. The study also offers actionable recommendations for healthcare institutions, emphasizing improved access to protective equipment, targeted psychological support, and resilience training.

By contributing to the literature on coping mechanisms and laying a foundation for future research, this study underscores the importance of institutional responsibility in supporting healthcare professionals during public health crises.

These results underscore the importance of targeted support mechanisms not only for healthcare workers but also for professionals in high-stress environments, such as social educators and psychologists. Future training programs in these fields could integrate coping strategies and resilience-building interventions to enhance competency development in stressful contexts.

The results highlight the psychological toll of the COVID-19 pandemic on healthcare workers, emphasizing the need for institutional support to mitigate stressors and promote effective coping strategies. Interventions should focus on enhancing access to protective equipment to alleviate fears of infection, on providing tailored psychological support for specific stressors, such as concerns for family members, and encouraging adaptive coping mechanisms, particularly for younger and less experienced healthcare workers.

This analysis underscores the critical importance of addressing the mental health needs of healthcare professionals during public health crises.

## 5. Conclusions

This study reveals the significant and varied psychological impact of the COVID-19 pandemic on healthcare workers. Elevated emotional distress was observed, largely attributed to perceived threats to family well-being and inadequate personal protective measures. Furthermore, coping strategies were found to diverge along professional lines, with nurses exhibiting a preference for emotion-focused coping mechanisms (e.g., religion, venting), while physicians gravitated toward problem-focused approaches (e.g., planning, active coping). These findings underscore the necessity of targeted psychological interventions tailored to the specific needs and stressors of different healthcare professions. Future research should explore the long-term mental health consequences of the pandemic on healthcare workers and evaluate the efficacy of tailored support programs in mitigating these effects.

## Figures and Tables

**Table 1:** Socio-demographic description of the sample.

Variable	Category	N = 338	Percentage (%)
Gender	Male	70	20.7
	Female	268	79.3
Residence	Urban	83	83.7
	Rural	55	16.3
Age groups	20–29	64	18.9
	30–39	73	21.6
	40–49	104	30.8
	50–59	61	18.0
	60–69	36	10.7
Profession	Nurse	133	39.3
	Physician	205	60.7

**Table 2:** Distribution of variables related to lifestyle.

Variable	Category	N = 338	Percentage (%)	95% CI
Healthy Meal Schedule	Yes	234	69.2	(64.3, 73.8)
	No	104	30.8	(26.2, 35.7)
Sleep Schedule Change	Yes	191	56.5	(51.2, 61.7)
	No	147	43.5	(38.3, 48.8)
Average Sleep Hours	<6 h	120	35.5	(30.5, 40.8)
	6–8	191	56.5	(51.3, 61.8)
	>8	97	8.0	(5.3, 11.6)
Sleep Quality	Unsatisfactory	52	15.4	(11.7, 19.8)
	Satisfactory	124	36.7	(31.7, 42.0)
	Good	122	36.1	(31.1, 41.3)
	Very good	40	11.8	(8.4, 16.0)
Smoking	Yes	93	27.5	(22.7, 32.8)
	No	245	72.5	(67.2, 77.3)
Change in Smoking Habit	Smoked More	28	31.1	(22.0, 41.4)
	Smoked Less	19	21.1	(13.4, 30.4)
	Smoked the Same	43	47.8	(37.2, 58.5)
Regular Physical Activity (at least 3 h/week)	Yes	134	39.6	(34.3, 45.1)
	No	204	60.4	(54.9, 65.7)
Maintained Physical Activity	Yes	168	49.7	(44.2, 55.2)
	No	170	50.3	(44.8, 55.8)

**Table 3:** Distribution of variables related to the COVID-19 experience.

Variable	Category	N = 338	Percentage (%)	95% CI
Close Contact with COVID-19 Patients	Yes	166	49.1	(45.4, 56.4)
	No	172	50.9	(43.6, 54.6)
Indirect Contact with a Confirmed Case	Yes	100	56.5	(24.7, 34.9)
	No	238	43.5	(65.1, 75.3)
Contact with Family Before Case Confirmation	Yes	135	35.5	(34.7, 45.4)
	No	174	39.9	(46.1, 56.9)
Recommended Isolation Due to Exposure	Yes	69	20.4	(16.2, 25.2)
	No	269	79.6	(74.8, 83.8)
COVID-19 Testing Experience	Yes	240	71.0	(65.8, 75.9)
	No	98	29.0	(24.1, 34.2) (24.1, 34.2)

**Table 4:** Hierarchy of perceived stress triggers (based on mean scores).

Perceived Stress Factors	Mean (M)	Standard Deviation (SD)	95% CI
The thought of potentially infecting your family	3.36	0.86	(3.25, 3.47)
The thought that a minor error could lead to infecting yourself and those around you	3.20	0.84	(3.09, 3.31)
The uncertainty of when the pandemic will end	3.14	0.77	(3.04, 3.24)
The fact that it is a new disease with no specific treatment yet	3.14	0.83	(3.03, 3.25)
Hearing about a colleague being infected	2.81	0.90	(2.69, 2.93)
The thought that protective equipment is not effectively protecting you	2.80	0.95	(2.67, 2.93)
Perception of hospital measures being ineffective in preventing the spread of COVID-19	2.56	0.99	(2.43, 2.69)
Experiencing mild respiratory symptoms causing fear of being infected	2.25	0.99	(2.12, 2.38)

**Table 5:** Comparison of coping strategies between physicians and nurses.

Coping Strategy	Profession	N	M	SD	t	*p* Value
Active Coping	Physician	205	2.39	0.71	0.98	0.327
	Nurse	133	2.31	0.77		
Planning	Physician	205	2.13	0.80	1.56	0.119
	Nurse	133	1.98	0.93		
Positive Reframing	Physician	205	2.28	0.67	−0.39	0.697
	Nurse	133	2.31	0.73		
Acceptance	Physician	205	2.40	0.61	2.06	0.041
	Nurse	133	2.24	0.79		
Humor	Physician	205	1.61	1.01	1.49	0.137
	Nurse	133	1.44	1.04		
Religion	Physician	205	1.38	1.05	−4.21	0.000
	Nurse	133	1.86	1.01		
Using Emotional Support	Physician	205	1.48	0.90	−2.61	0.010
	Nurse	133	1.75	1.00		
Using Instrumental Support	Physician	205	1.55	0.84	−1.57	0.118
	Nurse	133	1.70	0.94		
Self-Distraction	Physician	205	1.94	0.84	0.96	0.336
	Nurse	133	1.84	0.87		
Denial	Physician	205	0.25	0.55	−4.37	0.000
	Nurse	133	0.59	0.87		
Venting	Physician	205	0.8	0.72	−2.19	0.029
	Nurse	133	1.06	0.85		
Substance Use	Physician	205	0.30	0.63	2.98	0.003
	Nurse	133	0.12	0.45		
Behavioral Disengagement	Physician	205	0.37	0.58	0.14	0.887
	Nurse	133	0.36	0.63		
Self-Blame	Physician	205	0.54	0.67	1.69	0.091
	Nurse	133	0.42	0.59		
Problem-Focused Coping	Physician	205	1.86	0.58	−1.47	0.143
	Nurse	133	1.96	0.69		
Emotional Coping	Physician	205	1.73	0.47	−0.56	0.575
	Nurse	133	1.76	0.58		
Avoidance Oriented Coping	Physician	205	0.68	0.38	0.33	0.745
	Nurse	133	0.67	0.43		

**Table 6:** Biserial correlation of personal COVID-19-related events with anger and anxiety levels.

Personal Events Related to COVID-19 (Yes = 1, No = 0)	Anger (r)	Anxiety (r)
Close contact with suspected or confirmed COVID-19 cases (examined, performed medical/surgical procedures)	0.010	−0.027
Indirect contact with a confirmed COVID-19 case	0.013	−0.029
Contact with family members during a period of potential exposure	0.089	0.041
Isolation recommendation due to exposure to a suspected/confirmed COVID-19 case	0.034	0.050
COVID-19 testing	0.033	−0.082

## Data Availability

The datasets used and/or analyzed during the current study are available from the corresponding author upon reasonable request.
